# Barriers and facilitators to tele-support psychotherapy versus standard in-person mental health services for youth (15–30 Years) with depression in Kampala District, Uganda

**DOI:** 10.1371/journal.pgph.0006657

**Published:** 2026-07-30

**Authors:** Jeremiah Mutinye Kwesiga, John Mark Bwanika, Benedict Akimana, Happy Banonya, Brenda Kabakaari, Sowed Mutumba, Sabrina Bakeera Kitaka, Musisi Seggane, Moffat Nyirenda, Etheldreda Nakimuli-Mpungu

**Affiliations:** 1 Medical Research Council, Uganda Virus Research Institute and London School of Hygiene and Tropical Medicine (MRC/UVRI/ LSHTM) Research Unit, Entebbe, Uganda; 2 The Medical Concierge Group/Rocket Health Africa, Kampala, Uganda; 3 Department of Pediatrics, College of Health Sciences, Makerere University, Kampala, Uganda; 4 Department of Psychiatry, College of Health Sciences, Makerere University, Kampala, Uganda; 5 Butabika National Referral Mental Hospital, Ministry of Health of Uganda, Kampala, Uganda; 6 SEEK Group Support Psychotherapy Initiative, Kampala, Uganda; 7 Africa Centre for Applied Digital Health, Kampala, Uganda; ICDDR: International Centre for Diarrhoeal Disease Research Bangladesh, BANGLADESH

## Abstract

Depression remains a critical mental health challenge among young people in low-resource settings, where financial, structural, and social barriers frequently limit care access. Digital approaches, including Tele-Support Psychotherapy (TSP), have emerged as promising, scalable alternatives to Standard in-person Mental Health Services (SMHS), yet evidence comparing their implementation remains limited. This study explored facilitators and barriers influencing the feasibility, acceptability and engagement of youths with TSP versus SMHS for depression treatment in Kampala District, Uganda. A qualitative phenomenological study was conducted among youth aged 15–30 years enrolled in a randomized controlled trial evaluating both interventions. Data were collected through semi-structured key informant interviews and focus group discussions with participants and lay counselors and analyzed using deductive thematic analysis guided by the Consolidated Framework for Implementation Research (CFIR). In the parent trial, 95 of 154 participants (61.69%) in the TSP arm and 15 of 146 (10.27%) in the SMHS arm engaged with their assigned intervention; these figures provide contextual background for these qualitative findings. Key facilitators for both interventions included strong social support networks and higher income levels. Technological challenges, such as unreliable mobile phone connectivity, hindered TSP, while high costs and limited awareness were barriers to SMHS. Government policies both supported trust in digital interventions and, in some cases, constrained access while lay-counselor attributes, including on-phone session flexible scheduling and rapport-building skills facilitated TSP. TSP presents a viable alternative to SMHS, particularly for youth facing financial and logistical barriers to accessing facility-based mental health services. However, optimizing its delivery requires addressing technological constraints, ensuring consistent government support, and integrating mental health literacy initiatives. Findings underscore the need for flexible and contextually tailored models that leverage technology and address individual and systemic barriers to enhance mental health service access in resource-constrained settings.

## Introduction

Depression remains a critical mental health challenge among youth globally, with alarming prevalence rates observed across all continents [1–3]. In Africa, the burden of mental health disorders is particularly acute, evidenced by the region’s disproportionately high suicide rates and limited access to psychosocial interventions at the primary healthcare level [4]. Uganda has an estimated prevalence of 30.2% for depression, a relatively high prevalence which can be attributed to its history of conflict, high disease burden (including HIV), and persistent economic hardships [5,6]These factors have contributed to depression rates that exceed global averages [1,5]

The COVID-19 pandemic further exacerbated this mental health crisis, particularly among youth. Lockdown measures, social isolation, job losses, and economic uncertainty created a perfect recipe for increased depression and anxiety [7–9]. This impact was especially pronounced in Sub-Saharan Africa (SSA), where the pooled prevalence of common mental disorders among youth was significantly higher compared to other regions [10,11].

In Kampala District, Uganda’s capital and most populous city, these challenges are compounded by a severe shortage of mental health resources. Although the district offers more mental health services than other regions of the country, these services are often not youth-friendly and remain largely inaccessible. The availability of specialized mental health facilities is limited, and there is a critical shortage of trained mental health professionals [12,13]. Additionally, the National Mental Health Referral Hospital primarily focuses on severe cases, leaving mild-to-moderate conditions and preventive mental health care largely unaddressed.

This creates a substantial gap in the care of young people with depression, who often face additional barriers such as stigma, financial constraints, and geographical limitations.

To address these gaps in mental health service delivery, digital interventions have emerged as promising solutions [14,15]. These interventions have shown potential for increasing access to mental health care, especially in resource-limited settings [16–18]. Evidence from low- and middle-income countries suggests that the adoption of digital health interventions has been uneven across health sectors. For example, a systematic review examining the use of digital health tools among health workers in India found that most digital interventions focused primarily on maternal and child health services, while applications targeting mental health were comparatively limited [19]. This pattern highlights the disproportionate adoption of digital technologies across health domains, with mental health remaining relatively underrepresented despite its substantial disease burden. Furthermore, many of the digital interventions identified in the review were implemented within facility-based healthcare settings, rather than community-based or youth-focused service delivery models [19]. Similarly, in a broader systematic review examining digital health interventions in comparable low-resource contexts, only a small proportion of the identified interventions specifically addressed mental health care [20]. Evidence from sub-Saharan Africa also suggests that awareness of digital health solutions does not necessarily translate into real-world utilization. For instance, a study conducted in South Africa reported that although participants were aware of digital mental health interventions, none had direct experience using such services. The authors emphasized the importance of developing culturally appropriate and contextually tailored digital mental health interventions to improve acceptability and uptake [21]. Together, these findings suggest that while digital health innovations are expanding globally, the implementation and uptake of digital mental health services remain limited in many low-resource settings. Aderinto et al. emphasize that effectively addressing these challenges requires digital health interventions to be carefully tailored to the specific needs, sociocultural realities, and contextual conditions of the communities they are intended to serve [22].

In response to these gaps and the surge in mental health problems among youth during the COVID-19 pandemic, our research team adapted the evidence-based Group Support Psychotherapy (GSP) intervention into a mobile phone–delivered format known as Tele-Support Psychotherapy (TSP) culturally adopted to community. This adaptation aimed to leverage widely available mobile phone technology to improve access to psychological support among youth who may face barriers to facility-based mental health services. By examining barriers and facilitators to engagement with this tele-psychotherapy model in comparison with standard in-person mental health services, the present study contributes implementation insights into how digital mental health interventions can be delivered and utilized in a low-resource urban African context. In the parent 12-month pilot study, engagement with TSP was higher than with standard mental health services (SMHS), with 95 participants (61.7%) attending TSP sessions compared to 15 participants (10.3%) who sought in-person services [23]. These figures are presented solely to provide contextual background for the qualitative component. This data demonstrates the potential of TSP to improve engagement with mental health care in resource-constrained environments. Furthermore, evidence on temporal patterns of suicide attempts reveals that vulnerability peaks during specific hours, precisely when traditional in-person mental health facilities are often closing or understaffed [24]. This temporal gap underscores that flexible interventions like TSP are not merely convenient alternatives but potentially life-saving services capable of reaching individuals during these identified high-risk windows.

However, despite the promising results of TSP and other tele-psychotherapy approaches, traditional in-person mental health services are predominant services offered by both public and private providers despite limited uptake by youths. This discrepancy highlights the need to understand the factors influencing the adoption of these different service delivery models. In Kampala, the specific barriers and facilitators to tele-psychotherapy and in-person mental health services for youth with depression remain largely unexplored. Understanding these factors is crucial for designing and implementing effective mental health interventions tailored to the local context.

Therefore, this study aimed to identify and compare the perceived barriers and facilitators to accessing tele-psychotherapy and in-person mental health services among youth (15–30 years) with depression in Kampala District. We addressed the following research questions:

1. What are the perceived barriers and facilitators to accessing tele-psychotherapy among youth with depression in Kampala District?
2. How do these barriers and facilitators compare to those associated with in-person mental health services?

The findings from this study are intended to provide critical insights for policymakers, mental health practitioners, and researchers in designing, implementing, and scaling up mental health interventions for youth with depression in resource-limited settings.

## Methods

### Study design

This was a framework-guided qualitative descriptive study that explored the experiences of youth with depression who received and lay counselors who provided, psychotherapy through mobile phones and/or standard in-person counseling during a randomized controlled trial (RCT) carried out in Kampala, Uganda [23]. To comprehensively evaluate the implementation of TSP within this context, we employed the Consolidated Framework for Implementation Research (CFIR) [25]. The CFIR is a widely used framework that guides the in-depth assessment of factors influencing the implementation of health innovations. It consists of five domains: Intervention Characteristics, Outer Setting, Inner Setting, Characteristics of Individuals, and Process [25].

Guided by the CFIR, this study aimed to identify and understand the multi-level factors affecting the implementation of TSP and compare them with those associated with standard mental health services (SMHS). We chose a descriptive approach as it allows for a rich exploration of individuals’ lived experiences, enabling us to gain deeper insights into the barriers and facilitators influencing the adoption and use of both TSP and SMHS [26,27]. We anticipated that this approach would generate contextualized, evidence-based data crucial for designing and implementing effective mental health programs in similar resource-limited settings [28].

Semi-structured key informant interviews (KIIs) and focus group discussions (FGDs) were conducted between March and July 2024. The study focused on youth and emancipated minors, as they constitute over 55% of the population in urban centers in Uganda and are disproportionately affected by mental health challenges due to socioeconomic hardships and stigma [29,30].

The TSP model used in this study was adapted from the GSP model, a culturally sensitive intervention that has demonstrated effectiveness in managing depression among adults with HIV [31]. We made several key modifications to optimize GSP for remote delivery via mobile phones. These included adapting the session content and structure to maintain engagement and minimize distractions and recognizing the unique challenges of tele-psychotherapy. We also developed a comprehensive training program to equip lay counselors with the skills and knowledge necessary to deliver TSP effectively, ensuring fidelity to the model while accommodating the remote format. Additionally, we integrated technology-assisted tools, such as SMS reminders and mobile-based self-help resources, to enhance participant engagement and provide support between sessions. This adapted TSP model has proven feasible for managing depression and was well-received in the communities where it was implemented [23]. Using lay counselors to deliver TSP further amplifies its potential to bridge the substantial mental health support human resource gap prevalent in LMICs, offering a scalable and cost-effective solution.

### Study setting

This qualitative study was conducted in three communities within Kampala, Uganda: Kamwokya, Naguru, and Makerere. These communities represent three of the main administrative divisions of Kampala Central, Nakawa, and Kawempe divisions, respectively. Kampala, the capital city of Uganda, is made up of five distinct geographical divisions. Kampala is the most densely populated city in the country having over 2.5 million daytime inhabitants, with over 40% of these individuals staying in slums [29,32]. These slums are at increased risk for socioeconomic and public health-related challenges such as high rates of unemployment, substance abuse, poor access to healthcare services, including mental health and insecurity [33].

### Study population

Study participants and lay counselors (LCs) who had been assigned to receive and deliver psychotherapy respectively over mobile phones and/or standard mental health services were called for exit Key-Informant Interviews (KIIs) and Focus Group Discussions (FGDs). The study participants in this study were part of the 300 participants in the RCT whose details are published elsewhere [23].

After 12 months, purposive sampling was used to select the participants for the exit KIIs from participants who had completed and those who had not completed sessions in both the cases and the controls. Participants were selected from Kamwokya, Naguru, Key population, and Makerere communities aged between 16–30 years. The FGDs were gender-specific, ensuring both male and female perspectives were represented. Gender-specific groups allowed for open and comfortable discussions, particularly on sensitive topics like depression, stress, and anxiety. These groups were designed to include 6–10 participants, likely to maintain an optimal size for productive discussions [34].

### Sampling strategy

Purposive sampling was used to capture a diverse range of experiences with both intervention modalities and levels of engagement with psychotherapy. Two complementary qualitative data collection methods were employed: focus group discussions (FGDs) and key informant interviews (KIIs).

FGDs were conducted with youth participants who had been enrolled in the randomized trial. To encourage open discussion on sensitive mental health topics and to ensure balanced representation of perspectives, FGDs were stratified by gender. Each group consisted of approximately 6–10 participants.

KIIs were conducted with selected participants and lay counselors to obtain more in-depth individual perspectives on the implementation and delivery of the interventions. Participants for KIIs were purposively selected to capture variation in intervention modality (TSP vs. SMHS) and engagement status, including individuals who completed psychotherapy sessions and those who discontinued or did not complete the intervention. This approach allowed exploration of factors influencing both engagement and non-engagement with the interventions.

In total, six FGDs and nine KIIs were conducted during the study exit phase.

### Data collection

The KIIs and FGDs were designed to elicit in-depth information about the participants’ experiences with TSP and SMHS, guided by the CFIR domains. The interview guide was structured to explore factors related to user satisfaction with both interventions, including perceived benefits, challenges, and preferences (Intervention Characteristics). We also investigated external factors influencing service utilization, such as community attitudes towards mental health and the availability of alternative services (Outer Setting). Additionally, we examined structural factors within the implementing organizations, such as resource availability and staff attitudes towards technology (Inner Setting). Individual-level factors influencing service uptake and engagement, such as technology literacy, motivation, and prior experiences with mental health care, were also considered (Characteristics of Individuals). Finally, we explored implementation-related factors, such as communication strategies, training of lay counselors, and the fidelity of intervention delivery (Process).

The interviews and focused group discussions were facilitated by the study’s lead research coordinators (BH and BK). By the fourth FGD data saturation was reached, and no new codes were generated. However, given that our groups were gender-specific, we intended to have an upper limit of the number of FGDs [6] to achieve saturation, as recommended by different qualitative data collection literature [35–37]. Data saturation was defined as the point at which no new codes or themes relevant to the CFIR constructs emerged from successive focus group discussions. During the coding process, the research team observed that by the fourth FGD, no additional codes were generated beyond those already identified in the developing coding framework. Two additional FGDs were conducted to confirm thematic completeness and ensure adequate representation of both male and female participant groups. These focus group discussions were audio recorded, with the research assistants writing field notes to contextualize the transcripts during the analysis. This practice has been shown to improve the interview process and the subsequent data analysis and is a great supplement to the use of audio-recorded data alone [38,39]. The audio recordings were transcribed verbatim, and the interviews in Luganda were concurrently translated into English for analysis. The research assistant’s coordinator, HB, reviewed the transcribed interviews against the audio recordings to ensure consistency before final approval for analysis. The study participants were reimbursed approximately 5 dollars each for their time and participation in the interviews at the end of the interviews.

### Data analysis

Approved interview transcripts were uploaded to qualitative analysis software (MAXQDA v22.5.0). This study employed deductive thematic analysis, guided by the Consolidated Framework for Implementation Research (CFIR), to identify and categorize barriers and facilitators of TSP and SMHS. The transcripts were pre-coded based on the five CFIR domains, with two coders (HB and JMK) independently reviewing the data and assigning excerpts to the relevant CFIR category. Any discrepancies were resolved through discussion with the senior researchers (JMB and EN). This structured approach ensured that findings were systematically mapped to well-established implementation science constructs, facilitating a deeper understanding of the factors influencing mental health service delivery in resource-limited settings. Using the CFIR framework allowed for a systematic and comprehensive analysis of the barriers and facilitators to TSP and SMHS implementation. By mapping our findings to the CFIR constructs, we aimed to provide a more nuanced understanding of the factors influencing mental health service delivery in resource-limited settings, ultimately informing the development and implementation of more effective interventions.

## Results

### Contextual background from the parent trial

Baseline characteristics of participants in the parent randomized controlled trial were examined to provide contextual background for the present qualitative analysis (*Table A and Table B in* S1 Table).

Within the Tele-Support Psychotherapy (TSP) arm, participants who engaged with at least one session were broadly comparable to non-engagers across most psychosocial and clinical measures, including baseline anxiety, perceived stigma, alcohol use, social support, self-esteem, social group membership, and total social resources. Engagers tended to be slightly older and more likely to be employed, and there was a trend toward higher monthly income among engagers. Educational attainment was similar across groups. Gender differences in engagement were observed descriptively, with higher participation among females compared to males, while engagement among LGBTQ participants was comparatively low.

Similarly, within the Standard Mental Health Services (SMHS) arm, engagers and non-engagers were generally comparable across most socio-demographic and psychosocial indicators. Differences were observed descriptively in economic indicators, with engagers reporting relatively higher monthly income and savings. Other characteristics—including anxiety, perceived stigma, alcohol use, social support, self-esteem, and overall social resources—were largely similar between groups.

These baseline comparisons are presented to contextualize participant experiences explored in the qualitative component. The present study does not draw inferential conclusions from these quantitative differences but instead focuses on participants’ perceptions of barriers and facilitators to engagement.

### Barriers and facilitators to tele-support psychotherapy versus in-person mental health services

Of the 248 study participants who completed the 12-month follow-up assessments, 140 also completed the Exit survey. This survey explored barriers and facilitators to engagement with both tele-support psychotherapy (TSP) and standard mental health services (SMHS). To comprehensively analyze these factors, we employed the Consolidated Framework for Implementation Research (CFIR) [23]. The CFIR, with its five domains (Intervention Characteristics, Outer Setting, Inner Setting, Characteristics of Individuals, and Process), allowed us to systematically categorize the barriers and facilitators identified through our survey data. These findings are presented in Table A and Table B in S2 Table, offering a structured overview of the factors influencing engagement with TSP and SMHS.

Engagement with TSP and SMHS mental health interventions was influenced by various factors across multiple domains. Regarding intervention characteristics, TSP faced barriers such as long call-waiting times and occasional unintended costs. At the same time, facilitators included a toll-free platform, culturally appropriate therapy, and remote continuity of care. For SMHS, the lack of mental health providers and high transport costs posed challenges. Restrictive government policies, criminal prosecution, and societal stigma hindered access, with some participants requiring spousal approval to engage in psychotherapy sessions, which affected participation in either intervention. Nonetheless, informal support from friends, family, and spiritual guidance helped facilitate engagement. The inner setting presented challenges such as poor communication infrastructure, scheduling conflicts, and unreliable mobile access, though nearby counseling services and reliable communication devices improved participation. The implementation process faced outreach gaps, including missed information, leading to low attendance. Nonetheless, partnerships with trusted institutions like Makerere University improved trust, while a well-structured recruitment strategy ensured better awareness of available services.

## Discussion

Our previous study revealed a striking disparity in engagement rates between TSP and SMHS among youth with mild to moderate depression in Kampala. A remarkable 62% of participants engaged with TSP, compared to 10% who engaged with SMHS. This sparked our curiosity and led us to delve deeper into understanding the factors influencing young people’s access to mental health care in this setting.

This study compared facilitators and barriers to TSP and SMHS for youth experiencing depression in low-resource settings, utilizing the Consolidated Framework for Implementation Research (CFIR) to guide our investigation.

### Innovation domain

In this low-resource urban setting, TSP was perceived as offering several advantages over standard in-person services, including reduced travel demands and greater privacy—features that were particularly important given socioeconomic constraints and the prevailing policy environment. TSP was associated with higher engagement (62%) compared to SMHS (10%), especially among sexual minority youth who described avoiding facility-based care due to concerns about confidentiality and potential legal repercussions.

Technological issues, such as platform malfunctions and dropped calls due to poor network connectivity, further hampered service delivery, emphasizing the importance of reliable technology and contingency plans. These implementation challenges underscore the need for thorough training and support for both participants and counselors, clear communication channels, robust supervision and quality control measures, and investment in reliable technology to successfully deliver mental health interventions in similar settings.

### Outer setting domains

Our findings highlight the critical role of facilitators, particularly income that appeared to influence engagement with both TSP and SMHS. This observation which cut across both service delivery models underscores the pervasive influence of socioeconomic factors on mental health access and utilization. This aligns with existing literature demonstrating the association between poverty and mental health disparities [40,41]. For TSP, this may be attributed to the costs associated with technology, data plans, and reliable internet access, which can be prohibitive for low-income families. For SMHS, income may influence the ability to afford transportation to clinics or time off from work. Financial barriers must be addressed to ensure equitable access to mental healthcare, regardless of the delivery model.

Our study also uncovered distinct contextual challenges in Uganda, notably the disruption caused by government policies and anti-LGBTQ+ legislation, demonstrating how political and social instability can significantly impact mental health care delivery, particularly digital interventions reliant on stable technological infrastructures. Shortly before the implementation phase of this study, legislation was enacted in the study setting criminalizing same-sex relationships, including mandatory reporting requirements and severe legal penalties. Within this context, participants who identified as sexual minorities described heightened concerns about safety and confidentiality when seeking in-person health services. Several reported avoiding physical health facilities due to fear of disclosure or potential legal repercussions, and instead described relying more heavily on digital interventions. However, participants also noted that digital platforms presented their own challenges. Issues such as mandatory phone registration and poor connectivity underscored practical barriers associated with digital health implementation in low- and middle-income countries (LMICs), highlighting the necessity for adaptable strategies to mitigate unforeseen disruptions [42].

The strong influence of family and spousal support on engagement with both TSP and SMHS emphasizes the importance of social support in mental health-seeking behavior. This resonates with previous research highlighting the protective role of social support in mitigating the impact of mental health challenges [43,44]). Our findings suggest that individuals with strong social networks are more likely to initiate and maintain engagement with mental health services, regardless of the delivery format. This highlights the potential for leveraging social support networks in mental health interventions. Our findings indicated that peer support was associated with greater access to TSP when participants encountered barriers to facility-based services. Friends often shared information about toll-free lines and community counselors, suggesting that informal networks played an important role in navigating access challenges and highlighting the value of community-based resources and a strong motivation to address mental health needs. Future research should focus on developing and evaluating strategies to integrate TSP effectively within existing healthcare systems, ensuring that it complements and enhances, rather than replaces, existing services [42,44–46].

### Inner setting domain

A strong network of partners facilitated the provision of both TSP and SMHS. Our team included university researchers, tech experts from a digital health company, skilled clinicians from mental health facilities (Mulago, Butabika, Naguru), government folks, and local NGOs like SEEKGSP. The academic researchers designed and implemented the study, while the digital health company designed and supported the TSP platform. The clinicians brought their expertise in mental health care, and SEEKGSP identified and trained our lay counselors and provided ongoing support through the SEEKGSP Academy.

### Individual and implementations domain

We also learned the importance of flexibility and sustained engagement. Many individuals struggled to attend sessions consistently due to work, travel, or family demands, and some discontinued therapy once they felt better. However, continuing treatment beyond initial symptom relief is crucial to fully resolve depression and prevent relapse [47]. To maintain long-term engagement, mental health services must adapt to patients’ lives – for instance, by offering flexible appointment times and reinforcing the value of ongoing attendance even when symptoms improve. By making therapy more accessible and relevant to patients’ daily realities, we can bolster adherence and achieve more enduring outcomes in these vulnerable communities.

### Overall reflections and implications

These findings remind us that mental health care needs to be accessible and affordable, especially for young people in low-resource settings. Investing in digital tools like TSP, strengthening community support, and addressing financial barriers can make a real difference in the lives of those who need it most. We also need to keep working on making TSP a seamless part of the existing mental health system, ensuring it complements and enhances existing services.

We observed that profound socioeconomic hardships made it challenging for participants to prioritize mental health care. Many lived in poverty, which created obstacles to engaging with TSP and SMHS. For example, some lacked personal phones or lost them to theft, hindering their ability to connect with counselors. Others were intermittently detained for minor offenses, leading them to miss therapy sessions. These challenges mirror known disparities in mental health care access: patients with low income are at higher risk of dropping out of treatment [48], and “digital poverty” – lack of access to phones or internet – can impede participation in technology-based interventions [49].

Helping people in such communities effectively requires addressing these social challenges alongside clinical care. This might involve providing financial or logistical support, alternative means of counselor contact (e.g., community phones or in-person outreach), and advocating for policies that foster a more stable, supportive environment. Simply offering therapy is not enough; improving everyday living conditions is essential for better mental health outcomes [50].

Our findings are consistent with prior research in similar settings. Just like Sharma and Tyszka found in their work, we saw how important it is for people to be able to afford and access technology if they’re going to use digital mental health tools [51]. They also showed how vital it is to make these tools accessible to everyone, especially those who are often left behind – something that resonated with the challenges our participants faced in accessing both TSP and SMHS. And, like Araya and team discovered in Brazil and Peru, family and friends played a significant role in helping our participants engage with therapy [52]. Their research highlighted how involving families can make a real difference, which we also saw in our study—strong connections help people stick with mental health support, whether it’s TSP or SMHS.

This study has limitations. While our qualitative approach provided rich insights, it is inherently subjective, potentially limiting the generalizability of the findings. Despite efforts to ensure thoroughness, results might not apply to different populations or settings beyond Kampala’s urban slums. Additionally, although the Consolidated Framework for Implementation Research (CFIR) guided our analysis, its complexity could have obscured subtle contextual nuances [53]. Further limitations include reliance on self-reported data and susceptibility to biases such as recall and social desirability [54]. Finally, focus group discussions were also not stratified by engagement status, limiting direct comparison of experiences between these subgroups; however, quantitative data from the parent trial have been provided in the supplementary materials for additional context.

To advance the understanding of digital mental health interventions, future research should adopt broader and more rigorous designs. Investigations should encompass diverse populations and contexts, employing mixed-methods and cluster randomized controlled trials (RCTs), and integrating alternative frameworks like RE-AIM to capture comprehensive implementation outcomes [55]. Prioritizing analyses of mediating mechanisms and subgroup responsiveness will clarify for whom and how TSP is most effective [56]. Moreover, incorporating cost-effectiveness evaluations will be crucial for informing policy and resource allocation, ultimately enhancing mental health care accessibility and outcomes for youth in resource-constrained environments [57].

## Conclusion

This study highlights the multifaceted factors influencing access to TSP and SMHS among young people experiencing depression in Kampala, Uganda. We observed higher engagement with TSP compared to SMHS, and qualitative findings revealed that participation was shaped by individual-level factors such as income, social support, and digital literacy, as well as broader structural socioeconomic conditions. Barriers such as phone loss, network instability, and financial insecurity reflected wider contextual constraints rather than limitations of the intervention itself. These findings underscore the necessity of addressing structural socioeconomic challenges alongside innovation-specific barriers when implementing digital mental health interventions in low-resource settings. Integrating these considerations into program design such as providing phone credit support, strengthening digital infrastructure, and culturally tailoring intervention content may improve the feasibility, equity, and sustainability of mental health services for vulnerable youth.

## Supporting information

S1 Table Table A: Comparison of Baseline Characteristics between Tele-support psychotherapy (TSP) Engagers (N=95) and Non-Engagers (N=59). **Table B:** A comparison of baseline characteristics between those who engaged with standard mental health services (SMHS) by attending at least one session (Engagers, N = 15) and those who did not (Non-Engagers, N = 131).
(XLSX)

S2 Table Table A: Facilitators and barriers of tele-support psychotherapy based on the CFIR framework. **Table B:** Barriers and facilitators to engagement and completion of In-Person Therapy at Standard Mental Health Service Facilities based on the CFIR framework.
(XLSX)

S1 Text Semi-structured qualitative interview guide used to evaluate the acceptability, accessibility, and impact of tele-psychotherapy services. (DOCX)

S2 Text Coding framework illustrating themes and subthemes identified from post-intervention interviews. (DOCX)
