# Supplementary material for: Barriers and facilitators to tele-support psychotherapy versus standard in-person mental health services for youth (15–30 Years) with depression in Kampala District, Uganda
Source: PLOS Glob Public Health. 2026 Jul 30;6(7):e0006657. doi: 10.1371/journal.pgph.0006657 (PMC13422828; doi:10.1371/journal.pgph.0006657)
Supplement: S1 Text — (DOCX) [file pgph.0006657.s003.docx]

Exit Interview Guide

The Effect of Tele-Support Psychotherapy Delivered by TrainedLay Health Workers for Depression Treatment among Youth in Kampala District during the Covid-19 pandemic.

1) Welcome, consent process, and introduction

Welcome

*Good morning/afternoon/evening and welcome to our interview.*

*Thanks for taking the time to talk to us about your experience of using the Kampala Community Tele-psychotherapy Service. Your thoughts and opinions will be important in helping to improve services for members of the community. During the next hour or so, I would like to get your thoughts about mental health and using the service.*

*My name is ______________and assisting me is ____________.*

The goal of this study is to evaluate and improve the tele-psychotherapy service.

Tele-psychotherapy is a form of talk therapy delivered to people via the phone. During the talks, participants learn how to seek emotional & social support, learn positive coping skills and income-generating skills.

Today we would like to hear about your experience participating in the current tele-psychotherapy pilot. In particular, we seek your ideas and opinions about the service and your experience using it. We would like you to help us make the project better.

You are the experts: we learn from you (positive and negative). Caregivers/parents of individuals with chronic and complex health conditions are being invited to participate because you have important knowledge about particular experiences, needs, or perspectives that we hope to learn more about due to our discussion today.

Consent

We will go over the informed consent form before we start our interview/focus-group discussion (FGD) to be sure that you understand why we have this interview and to be sure that you voluntarily want to participate.

2) Interview/ FGD logistics and ground rules

Logistics

- The meeting will last about one hour (60 minutes)
- Feel free to move around.
- Where is the bathroom? Exit?
- Help yourself to refreshments

Ground rules

- Please feel free to share your ideas and opinions, this is intended as an informal conversation where we can understand more from your perspective.
- There are no right or wrong answers to questions – just ideas, experiences, and opinions, which are all valuable
- If you agree to it, the session will be audio recorded to help us gather more detailed information about your responses. We will also take handwritten notes, which will allow us to double-check our data for accuracy.
- Enjoy the discussions

Any questions?

| Date |  |
| --- | --- |
| Interview number |  |
| Researchers note (for memory) |  |
| Beneficiary Group |  |
| Age |  |
| Gender |  |
| Education level |  |
| Region of birth |  |
| Region of residence |  |
| Civil State | a) single; b) married; c) divorced; d) cohabiting; e) widow |
| Economic Activities |  |

Post-Intervention Interview Questions

The questions listed below are intended as prompts for an open, one-on-one conversation. Additional questions can also be included according to the responses.

1. User Satisfaction: to gain general feedback about using the KCTP service
2. How did you first hear about the tele-psychotherapy service?

?

1. Tell me all about your experience using the tele-psychotherapy service?
2. What did you expect from using the service?
3. Did the service meet your initial expectations?
4. What effect did the intervention have on your life?
5. In your opinion, did the service help to improve your mental health problems? Please give examples.
6. Which sessions were most helpful? Why?
7. Which sessions were not helpful? Why?
8. Which topics would you have liked to discuss more?
9. What would have made it more interesting for you?
10. What would stop you from using the service again?
11. How likely would you be to recommend the service to other people?
12. What would make people more likely to use the service?
13. What would prevent people from using the service?
14. How would you improve the service?
15. Is there anything else you would like to discuss?
16. Digital use and access: to identify barriers to accessing the KCTP, and how they are negotiated
17. How did you access the service?
    1. Probe on type of mobile phone or smartphone and how it was accessed.
18. Does the phone you called with belong to you?
    1. If not, who did you borrow it from? How was it for you to ask to borrow their phone for the therapy session? Would they ever refuse to lend the phone? If so, for what reason?
19. Even though the service is free of charge, were there any additional financial costs incurred to use the service?
    1. E.g. probe on time off work, charging, data costs.
20. Have you used your phone at any other time to access health information or a health service?
    1. If yes, please give examples. Which platforms do you use? (e.g. phone calls, WhatsApp, Google, other telecare services or mHealth apps)
21. How did you find the timing and frequency of the calls?
    1. Were they too short / long / (in)frequent?
    2. Probe on work schedules
22. Do you have any ideas for making access to the service easier?
23. Privacy and confidentiality: to understand perceptions about the community-based approach and prevent any issues related to privacy and confidentiality
24. Where did you make the phone call from?
25. Were there any issues with finding a private space for the call?
26. How did you feel discussing personal matters over the phone?
27. How did you feel discussing personal matters with the Lay Counsellor?
28. Did you feel that the service providers guaranteed privacy and confidentiality?
29. Were there any issues you would like to address?
30. Do you have any ideas for improving the privacy of the service?
