# Supplementary material for: Barriers and facilitators to tele-support psychotherapy versus standard in-person mental health services for youth (15–30 Years) with depression in Kampala District, Uganda
Source: PLOS Glob Public Health. 2026 Jul 30;6(7):e0006657. doi: 10.1371/journal.pgph.0006657 (PMC13422828; doi:10.1371/journal.pgph.0006657)
Supplement: S2 Text — (DOCX) [file pgph.0006657.s004.docx]

Code System

| **Code System** | **Memo** | **Frequency** |
| --- | --- | --- |
| Code System |  | 200 |
| Prior experience of digital health access |  | 5 |
| Drivers for Telepyschotherapy Use |  | 3 |
| Accesibility to phones for communication |  | 8 |
| Willingness to recommend to other people |  | 6 |
| Mode of recommendation |  | 1 |
| Reason for recommendation |  | 3 |
| Therapy Sessions Experience |  | 1 |
| Privacy |  | 12 |
| Duration of Sessions |  | 8 |
| Therapeutic Impact |  | 7 |
| Client Expectations of Therapy Sessions |  | 15 |
| Behaviour Change |  | 13 |
| Initiation |  | 10 |
| Awareness of Telepyschotherapy |  | 1 |
| Source of information about service |  | 11 |
| Importance of instruction |  | 3 |
| Challenges |  | 0 |
| Unintended expenditures |  | 5 |
| Client Challenges |  | 5 |
| Privacy |  | 13 |
| Sheduling |  | 7 |
| System-related |  | 7 |
| Counselor-related |  | 6 |
| Recommendations |  | 0 |
| Reminders |  | 1 |
| Change of Counselors |  | 1 |
| Duration of calls |  | 1 |
| Telepyschotherapy sessions Continuation |  | 1 |
| System configuration |  | 1 |
| Face-to-face interactions |  | 2 |
| Less Impactful Session |  | 3 |
| Telepyschotherapy Impact |  | 8 |
| Learning avenue |  | 2 |
| Positive Counselor Attributes |  | 5 |
| Impactful Session |  | 11 |
| Gaining Trust |  | 10 |
| Opening Up |  | 4 |
