# Supplementary material for: Barriers and facilitators to tele-support psychotherapy versus standard in-person mental health services for youth (15–30 Years) with depression in Kampala District, Uganda
Source: PLOS Glob Public Health. 2026 Jul 30;6(7):e0006657. doi: 10.1371/journal.pgph.0006657 (PMC13422828; doi:10.1371/journal.pgph.0006657)
Supplement: Attachment — Submitted filename: Response to Reviewers.docx [file pgph.0006657.s007.docx]

Md. Alamgir Hossain, M.S.S., M.P.H.
Academic Editor
PLOS Global Public Health

Dear Dr. Hossain,

**RE: Response to Reviewer Comments**

Thank you for the opportunity to revise and resubmit our manuscript entitled “Barriers and Facilitators to Tele-Support Psychotherapy Versus Standard In-Person Mental Health Services for Youth (15–30 Years) with Depression in Kampala District, Uganda.” We are grateful to you and the reviewers for the thoughtful and constructive feedback provided on our manuscript.

We have carefully reviewed all comments and suggestions and have revised the manuscript accordingly. The revisions have substantially improved the clarity, methodological consistency, and overall presentation of the study. In particular, we addressed concerns related to the alignment of the analytic approach with the Consolidated Framework for Implementation Research (CFIR), clarified the qualitative study design, strengthened the methodological description of sampling and data saturation, reduced redundancy in the Introduction and Discussion sections, and refined the interpretation of findings to ensure appropriate qualitative framing.

We have also clarified the distinction between contextual quantitative information derived from the parent randomized controlled trial and the qualitative findings reported in this manuscript. To avoid methodological ambiguity, statistical comparisons previously presented in the main manuscript have been relocated to the Supplementary Material and are now described solely as contextual background information.

A detailed, point-by-point response to each reviewer comment is provided below. For transparency and ease of review, we indicate where changes have been made in the revised manuscript using page references.

We sincerely appreciate the reviewers’ insightful comments and the opportunity to improve our work. We hope that the revisions adequately address all concerns and that the manuscript is now suitable for publication in *PLOS Global Public Health*.

Thank you for your time and consideration.

Sincerely.

Etheldreda Nakimuli Mpungu, PhD

**Point-by-point Response to Reviewers’ Comments**

**REVIEWER 1**

**Major concerns:**
**Comment 1**. While the authors’ state that comparative assessments into in person and tele health services are scarce, there have been many papers exploring this area since the COVIC pandemic. For example, there have been publications on this area in PLOS digital health and PLOS GPH journals.
How is this study adding to the existing literature in this field?

***Response:*** *We thank the reviewer for this important observation. In the revised manuscript, we have incorporated evidence showing that while digital health interventions are expanding globally, adoption has been uneven across health sectors. Specifically, our added review highlights that mental health remains relatively underrepresented compared to areas such as maternal and child health, and that many interventions are facility-based rather than community- or youth-focused. By situating our study within this context, we clarify that our work adds to the literature by providing context-specific, youth-focused insights into tele-support psychotherapy (TSP) implementation in a low-resource urban setting in Uganda. Unlike previous studies that primarily document adoption or usage patterns, our study explores how structural, sociocultural, and intervention-specific factors interact to shape engagement, providing actionable guidance for implementing digital mental health interventions in similar LMIC contexts.*


**Comment 2.** A more in-depth literature review of papers on this topic is required, and the authors are requested to elaborate how their findings add to or differ from existing data from other developing countries. While a few papers have been referenced in the discussion section, there is a huge body of work in this area, and a more detailed justification of this work is required.
Many of the findings of this study: for example, low income, poor internet access, or lack of govt support being barriers to engagement can be readily anticipated as barriers in a resource limited setting and have been demonstrated in prior research in similar settings worldwide.

***Response****: We thank the reviewer for this insightful comment. In the revised manuscript, we incorporated additional literature from LMICs showing that while awareness of digital health solutions exists, real-world utilization remains limited, particularly for mental health services. Our literature review highlights that interventions often remain facility-based, with limited community- or youth-focused delivery, and that culturally appropriate, contextually tailored interventions are essential for uptake. Against this backdrop, our study adds value by documenting qualitative, context-specific experiences of youth engagement with tele-support psychotherapy (TSP) in Kampala, Uganda. While barriers such as low income, limited internet access, and lack of government support have been identified previously, our findings provide nuanced understanding of how these structural constraints interact with intervention-specific features—such as remote delivery via mobile phones and engagement strategies by lay counselors—offering practical lessons for designing and scaling youth-centered digital mental health services in resource-limited urban settings.*

3. The conclusions appear ambiguous and vague, with lack of specific results or implications.

***Response:*** *We thank the reviewer for this comment. The conclusion has been revised to more clearly highlight key findings of the study, including the higher engagement observed with tele-support psychotherapy (TSP) and the contextual barriers influencing participation. We have also clarified the practical implications for implementing digital mental health interventions in low-resource settings, particularly the need to address both structural socioeconomic constraints and intervention-specific barriers.*

Minor concerns:
**Comment:** While the authors have compared engagers vs non engagers in each group (tele health vs in person), were there common characteristics between engagers in both groups? And between the non engagers in both groups? This is important to identify factors that influence health care uptake regardless of the intervention used.

***Respond:*** *We thank the reviewer for this insightful comment and agree that comparing characteristics of engagers and non-engagers can provide valuable insights into mental health service uptake. However, the focus group discussions in this study were not stratified by engagement status, as they were designed to capture a broad range of participant experiences with tele-support psychotherapy (TSP) and standard mental health services (SMHS). To provide additional context, we have included quantitative data from the parent trial comparing engagers and non-engagers in the supplementary materials. We have also acknowledged this as a limitation in the revised manuscript and note that future studies could benefit from stratifying qualitative data collection by engagement status.*

**SREVIEWER TWO**

**Overall Assessment**

Topic is timely, policy-relevant, and of substantial global health interest. The integration of implementation science (CFIR) with qualitative inquiry is commendable. However, the manuscript presents conceptual inconsistencies, methodological ambiguities, structural issues, and interpretational concerns that require careful revision prior to publication.

**Abstract**

**Comment:** The abstract states “inductive thematic analysis,” whereas the Methods section later specifies deductive thematic analysis guided by CFIR. This contradiction must be resolved.

**Response:** *We thank the reviewer for highlighting this important inconsistency. The initial reference to “inductive thematic analysis” in the abstract was an oversight and does not accurately reflect the analytic approach used in the study. As described in the Methods section, the analysis was primarily deductive and guided by the Consolidated Framework for Implementation Research (CFIR). We have revised the abstract to ensure alignment with the Methods section and to clearly state that a deductive thematic analysis informed by CFIR was conducted (Page 2).*

**Comment:** Precise engagement percentages (e.g., 61.69%, 10.27%) are presented without explanation of their analytical role within a phenomenological qualitative study. The distinction between RCT outcomes and qualitative findings should be clarified.

***Response:*** *We thank the reviewer for this insightful comment. We agree that presenting precise engagement percentages without clarifying their analytical role may create confusion within a phenomenological qualitative study. The engagement figures (61.69% and 10.27%) were derived from the parent randomized controlled trial and were intended solely to provide descriptive context regarding participant exposure to the respective interventions, not to serve as analytic outcomes of the qualitative component.*

*In response, we have revised the manuscript to explicitly distinguish between the quantitative RCT indicators and the phenomenological qualitative findings. The engagement percentages are now clearly described as contextual background data from the parent trial, and we have removed language that could imply inferential or outcome-based conclusions within the qualitative strand (Page 2).*

**Introduction**

**Comment:** The same numerical comparison between TSP and SMHS engagement appears multiple times. This risks redundancy and weakens narrative flow.

***Response:*** *We thank the reviewer for this observation. In the revised manuscript, the engagement comparison between tele-support psychotherapy (TSP) and standard mental health services (SMHS) is now presented as contextual background from the parent 12-month pilot study. All subsequent references to engagement patterns are now described narratively, without repeating the numerical percentages. These revisions reduce redundancy, improve narrative flow, and clearly distinguish quantitative context from the qualitative analysis.(Page 5)*

**Comment:** Statements such as Uganda ranking among “top countries with high depression rates” should be cautiously framed and directly supported by high-quality epidemiological sources.

***Response:*** *We thank the reviewer for this important observation. In the revised manuscript, we have removed the comparative statement suggesting that Uganda ranks among the countries with the highest depression rates. Instead, we now report a prevalence estimate from the literature indicating that depression affects approximately 30.2% of individuals in certain Ugandan populations, and we have framed this more cautiously to reflect variability across studies and populations. We also contextualize this burden by noting broader structural factors such as the history of conflict, the high burden of chronic diseases including HIV, and ongoing socioeconomic challenges. These revisions ensure that the statement is more appropriately supported and cautiously interpreted. (Page 4)*

**Comment:** The introduction justifies the need for teletherapy using the context of COVID-19 and resource shortages but overlooks temporal vulnerability—youth need support during specific vulnerable hours when clinics are closed. Adding this temporal aspect, supported by a recent study, fills this gap by establishing teletherapy as the only solution capable of reaching youth during these specific high-risk windows. Write below phrase with recent literature as suggested to strengthen the gap..

"Evidence on temporal patterns of suicide attempts reveals that vulnerability peaks during specific hours, precisely when traditional in-person mental health facilities are often closing or understaffed [Sheikh et al., 2024 Elsevier, <https://doi.org/10.1016/j.bbii.2024.100072> ]. This temporal gap underscores that flexible interventions like tele-support psychotherapy (TSP) are not merely convenient alternatives but potentially life-saving services capable of reaching individuals during these identified high-risk windows."

***Response:*** *We thank the reviewer for this insightful observation. We agree that the original Introduction emphasized structural and pandemic-related access barriers but did not sufficiently address the temporal dimension of vulnerability among youth with depression. In response, we have incorporated recent evidence highlighting temporal patterns of suicide attempts, which demonstrate that vulnerability peaks during late evening and night-time hours—periods when traditional in-person mental health services are often closed or understaffed (Sheikh et al., 2024).*

*We have revised the Introduction to explicitly acknowledge this temporal misalignment between peak psychological distress and service availability. (Page 5)*

**Methods**

**Comment:** The manuscript describes the study as: Phenomenological qualitative study, Guided by CFIRm Embedded within an RCT

Phenomenology is inherently inductive and experience-centered, whereas CFIR-based coding is typically deductive and framework-driven. The epistemological compatibility should be justified. Clarify whether the study is truly phenomenological or a framework-guided qualitative descriptive study.

***Response:*** *We thank the reviewer for raising this important epistemological concern. We acknowledge that phenomenology is traditionally inductive and centred on uncovering the essence of lived experience, whereas coding guided by the Consolidated Framework for Implementation Research (CFIR) reflects a deductive, framework-driven analytic orientation.*

*Upon careful reconsideration, we agree that the analytic approach employed in this study aligns more closely with a framework-guided qualitative descriptive design than with a strictly phenomenological methodology. While the study explored participants’ experiences, the analysis was primarily structured deductively using CFIR domains to examine implementation-related determinants.*

*Accordingly, we have revised the manuscript to remove references to phenomenology, clarify the study design as a qualitative descriptive study guided by CFIR, and ensure methodological consistency throughout the abstract, methods, and discussion sections. (Page 7)*

**Comment:** Earlier: “inductive thematic analysis” and later: “deductive thematic analysis guided by CFIR”

Both cannot simultaneously be true without methodological clarification.

**Response:** *We thank the reviewer for identifying this inconsistency. We agree that describing the analysis as both “inductive thematic analysis” and “deductive thematic analysis guided by CFIR” without clarification creates methodological confusion. The earlier reference to “inductive thematic analysis” was an oversight and did not accurately reflect the analytic approach undertaken.*

*We have revised the abstract, Methods section, and all related descriptions to consistently state that a deductive thematic analysis informed by CFIR was used. This correction ensures conceptual and methodological coherence throughout the manuscript. (Page 11)*

**Comment:** The sampling logic lacks sufficient detail: How many KIIs? How many FGDs per subgroup? How were engagers vs non-engagers balanced?

***Response:*** *We thank the reviewer for highlighting the need for greater clarity regarding the qualitative sampling strategy. We have revised the Methods section to provide explicit detail on the number and purpose of the qualitative interviews under a new added sampling strategy statement. In total, six focus group discussions (FGDs) and nine key informant interviews (KIIs) were conducted at study exit. FGDs were stratified by gender to facilitate open discussion of sensitive mental health topics, while KIIs were purposively selected to capture variation in intervention modality and engagement status (engagers vs non-engagers). (Page 9)*

**Comment:** The statement: “By the fourth FGD data saturation was reached” is acceptable but requires operational definition of saturation.

***Response:*** *We thank the reviewer for highlighting the need to clarify the operational definition of data saturation. In the revised manuscript, we have expanded this section to define saturation as the point at which no new codes or themes relevant to the analytical framework emerged from successive focus group discussions during the coding process. During analysis, the research team observed that by the fourth FGD, no additional codes were identified beyond those already present in the developing coding framework. Nevertheless, two additional FGDs were conducted to confirm thematic completeness and to ensure representation across gender-stratified groups. The Methods section has been revised accordingly to reflect this clarification. (Page 10)*

**Results**

**Comment:** Major Concern is Quantitative Tables in Qualitative Study

Tables 1 and 2 present statistical hypothesis testing (t-tests, χ² tests, p-values), which is inconsistent with the stated qualitative phenomenological design. Either integrate as a mixed-methods design Or relocate statistical comparisons to supplementary material

***Response:*** *We thank the reviewer for this important observation. We agree that the inclusion of statistical hypothesis testing (t-tests, χ² tests, and p-values) in the main manuscript may create the impression of a mixed-methods design, which does not accurately reflect the primary qualitative focus of this study.*

*The statistical comparisons presented in Tables 1 and 2 were derived from the parent randomized controlled trial and were intended solely to provide descriptive background characteristics of participants across study arms. They were not part of the analytic framework of the present qualitative investigation.*

*To avoid methodological confusion, we have relocated Tables 1 and 2 to the Supplementary Material and revised the main text to clarify that these data are provided for contextual purposes only. The primary manuscript now focuses exclusively on the qualitative findings guided by CFIR. (Page 12 and Supplementary Material)*

**Comment:** “Marginally significant difference (p=0.055)” Strictly speaking, this is not statistically significant and should be framed cautiously.

***Response:*** *We thank the reviewer for this clarification. We agree that describing a p-value of 0.055 as “marginally significant” may be misleading and risks overinterpretation.*

*However, in response to this and related methodological concerns, we have removed all statistical hypothesis testing language including references to p-values and statistical significance from the manuscript. The quantitative comparisons derived from the parent trial have been relocated to the Supplementary Material and are now presented solely as descriptive contextual information. The primary manuscript no longer characterizes any findings in terms of statistical significance.*

*These revisions ensure conceptual clarity and maintain the qualitative focus of the study.*

**Comment:** Large gender disparities (female vs male engagement) are important but insufficiently explored. Potential sociocultural explanations should be considered.

***Response:*** *We thank the reviewer for highlighting this important disparity that warrants deeper exploration. In light of the earlier guidance to minimize the presentation of statistical comparisons in the main manuscript, we have removed detailed quantitative emphasis on gender differences and instead integrated a more interpretive discussion of engagement patterns within the qualitative findings and Discussion section. More specifically, within the Outer Setting domain, spousal permission and partner influence emerged as important contextual factors shaping women’s ability to participate, reflecting prevailing household power dynamics and gendered decision-making structures. (Tables)*

**Comment:** Certain barriers (e.g., phone loss, network instability) could be analytically distinguished between intervention vs structural poverty.

*We thank the reviewer for this insightful observation. In the revised manuscript, we have clarified the distinction between barriers directly related to the implementation of tele-support psychotherapy (TSP) and those arising from broader structural and socioeconomic conditions. Specifically, factors such as phone loss, limited access to electricity for charging devices, and unstable network connectivity were interpreted as structural constraints reflecting the socioeconomic realities of participants living in resource-limited urban settings, rather than limitations inherent to the intervention itself. We have revised the Results and Discussion sections to more explicitly distinguish these structural barriers from intervention-specific implementation challenges, thereby providing a clearer interpretation of the contextual factors influencing the feasibility and delivery of TSP. (Tables)*

**Discussion**

**Comment:** The discussion is comprehensive but would benefit from greater analytical discipline and reduced narrative drift.

Several themes are reiterated multiple times (income, social support, poverty). Condensation would improve impact.

***Response:*** *We thank the reviewer for this helpful suggestion. In the revised manuscript, we have carefully reviewed the Results and Discussion sections to reduce redundancy and improve clarity. Repeated references to closely related themes—such as income constraints, social support, and broader poverty-related challenges—have been condensed and integrated where appropriate. This restructuring allowed us to present these interconnected issues more succinctly while preserving the analytical depth of the findings. As a result, the revised sections provide a clearer and more streamlined presentation of the key themes. (Page 14-18)*

**Comment:** The discussion occasionally implies causal inference (e.g., income “facilitates engagement”), which is not appropriate for qualitative design. Prefer like “Income appeared to influence engagement…”

***Response:*** *We appreciate this important clarification. We agree that some phrasing unintentionally suggested causal inference. Throughout the revised manuscript, we have replaced causal language with language more appropriate for qualitative research. These revisions ensure that the findings are framed as interpretive and experiential rather than causal, aligning more appropriately with the study design. (Pages14-18)*

**Comment:** Statements linking anti-LGBTQ+ legislation to engagement barriers are important but require careful evidentiary framing to avoid overgeneralization.

***Response:*** *We thank the reviewer for this important observation. We agree that our original phrasing risked implying broader population-level effects beyond what our qualitative data support. In the revised manuscript, we have carefully reframed this section to ensure that conclusions remain grounded in participant accounts rather than generalized claims.*

*Specifically, we now clarify that the observations regarding anti-LGBTQ+ legislation emerged from narratives of participants who identified as sexual minorities. We further note that increased reliance on digital interventions was described by some participants within this sociopolitical context, rather than asserting that the legislation directly caused disengagement from in-person care. (Page 15)*

**Comment:** Although CFIR guided analysis, the discussion is not fully structured around CFIR domains. Stronger framework integration would enhance theoretical contribution.
***Response:*** *We thank the reviewer for this insightful observation. In response, we have strengthened the integration of CFIR in the Discussion section. Each major theme is now explicitly mapped to the relevant CFIR domain.*

*This structured approach clarifies how identified barriers and facilitators align with established implementation constructs, enhancing the theoretical contribution of the study. By explicitly linking qualitative insights to CFIR, we provide a more systematic and conceptually grounded interpretation of factors influencing engagement with tele-support psychotherapy and standard mental health services. (Pages 14-18)*

**Comment:** “Our findings echo what others have seen…” Should be formalized.

***Response:*** *We agree and have revised this phrasing in the manuscript to adopt a more formal academic tone. This has been replaced with “are consistent with prior research” appropriate. This revision improves scholarly tone and precision. (Page 17)*

*s*

**Conclusion**

**Comment:** Suggestion for Strengthening: “These findings highlight the necessity of addressing structural socioeconomic constraints alongside innovation-specific barriers when implementing digital mental health interventions in low-resource settings.”

***Response:*** *We thank the reviewer for this insightful suggestion. In the revised manuscript, we have strengthened the conclusion to emphasize the importance of addressing both structural socioeconomic constraints and intervention-specific barriers when implementing digital mental health interventions in low-resource settings. This addition clarifies the broader implications of our findings for the design and implementation of tele-support psychotherapy and similar digital mental health innovations. (Page 19)*
