# Supplementary material for: Barriers and facilitators to tele-support psychotherapy versus standard in-person mental health services for youth (15–30 Years) with depression in Kampala District, Uganda
Source: PLOS Glob Public Health. 2026 Jul 30;6(7):e0006657. doi: 10.1371/journal.pgph.0006657 (PMC13422828; doi:10.1371/journal.pgph.0006657)
Supplement: Attachment — Submitted filename: Comments.docx [file pgph.0006657.s005.docx]

Overall Assessment

Topic is timely, policy-relevant, and of substantial global health interest. The integration of implementation science (CFIR) with qualitative inquiry is commendable. However, the manuscript presents conceptual inconsistencies, methodological ambiguities, structural issues, and interpretational concerns that require careful revision prior to publication.

Abstract

The abstract states “inductive thematic analysis,” whereas the Methods section later specifies deductive thematic analysis guided by CFIR. This contradiction must be resolved.

Precise engagement percentages (e.g., 61.69%, 10.27%) are presented without explanation of their analytical role within a phenomenological qualitative study. The distinction between RCT outcomes and qualitative findings should be clarified.

Introduction

The same numerical comparison between TSP and SMHS engagement appears multiple times. This risks redundancy and weakens narrative flow.

Statements such as Uganda ranking among “top countries with high depression rates” should be cautiously framed and directly supported by high-quality epidemiological sources.

The introduction justifies the need for teletherapy using the context of COVID-19 and resource shortages but overlooks temporal vulnerability—youth need support during specific vulnerable hours when clinics are closed. Adding this temporal aspect, supported by a recent study, fills this gap by establishing teletherapy as the only solution capable of reaching youth during these specific high-risk windows. Write below phrase with recent literature as suggested to strengthen the gap..

"Evidence on temporal patterns of suicide attempts reveals that vulnerability peaks during specific hours, precisely when traditional in-person mental health facilities are often closing or understaffed [Sheikh et al., 2024 Elsevier, <https://doi.org/10.1016/j.bbii.2024.100072> ]. This temporal gap underscores that flexible interventions like tele-support psychotherapy (TSP) are not merely convenient alternatives but potentially life-saving services capable of reaching individuals during these identified high-risk windows."

Methods

The manuscript describes the study as: Phenomenological qualitative study, Guided by CFIRm Embedded within an RCT

Phenomenology is inherently inductive and experience-centered, whereas CFIR-based coding is typically deductive and framework-driven. The epistemological compatibility should be justified. Clarify whether the study is truly phenomenological or a framework-guided qualitative descriptive study.

Earlier: “inductive thematic analysis” and later: “deductive thematic analysis guided by CFIR”

Both cannot simultaneously be true without methodological clarification.

The sampling logic lacks sufficient detail: How many KIIs? How many FGDs per subgroup? How were engagers vs non-engagers balanced?

The statement: “By the fourth FGD data saturation was reached” is acceptable but requires operational definition of saturation.

Results

Major Concern is Quantitative Tables in Qualitative Study

Tables 1 and 2 present statistical hypothesis testing (t-tests, χ² tests, p-values), which is inconsistent with the stated qualitative phenomenological design. Either integrate as a mixed-methods design Or relocate statistical comparisons to supplementary material

“Marginally significant difference (p=0.055)” Strictly speaking, this is not statistically significant and should be framed cautiously.

Large gender disparities (female vs male engagement) are important but insufficiently explored. Potential sociocultural explanations should be considered.

Certain barriers (e.g., phone loss, network instability) could be analytically distinguished between intervention vs structural poverty.

Discussion

The discussion is comprehensive but would benefit from greater analytical discipline and reduced narrative drift.

Several themes are reiterated multiple times (income, social support, poverty). Condensation would improve impact.

The discussion occasionally implies causal inference (e.g., income “facilitates engagement”), which is not appropriate for qualitative design. Prefer like “Income appeared to influence engagement…”

Statements linking anti-LGBTQ+ legislation to engagement barriers are important but require careful evidentiary framing to avoid overgeneralization.

Although CFIR guided analysis, the discussion is not fully structured around CFIR domains. Stronger framework integration would enhance theoretical contribution.

“Our findings echo what others have seen…” Should be formalized.

Conclusion

Suggestion for Strengthening: “These findings highlight the necessity of addressing structural socioeconomic constraints alongside innovation-specific barriers when implementing digital mental health interventions in low-resource settings.”
