# Supplementary material for: Barriers and facilitators to tele-support psychotherapy versus standard in-person mental health services for youth (15–30 Years) with depression in Kampala District, Uganda
Source: PLOS Glob Public Health. 2026 Jul 30;6(7):e0006657. doi: 10.1371/journal.pgph.0006657 (PMC13422828; doi:10.1371/journal.pgph.0006657)
Supplement: Peer review history

# Peer review: Decision Letter 0

**DOI:** 10.1371/journal.pgph.0006657.r001

**Authors:** Md. Alamgir Hossain

22 Feb 2026

PGPH-D-25-03671

Barriers and Facilitators to Tele-Support Psychotherapy Versus Standard In-Person Mental Health Services for Youth (15-30 Years) with Depression in Kampala District, Uganda.

PLOS Global Public Health

Dear Dr. Etheldreda Nakimuli,

Thank you for submitting your manuscript to PLOS Global Public Health. After careful consideration, we feel that it has merit but does not fully meet PLOS Global Public Health’s publication criteria as it currently stands. Therefore, we invite you to submit a revised version of the manuscript that addresses the points raised during the review process.

Please submit your revised manuscript by 14/03/2026. If you will need more time than this to complete your revisions, please reply to this message or contact the journal office at globalpubhealth@plos.org. When you're ready to submit your revision, log on to https://www.editorialmanager.com/pgph/ and select the 'Submissions Needing Revision' folder to locate your manuscript file.

Please include the following items when submitting your revised manuscript:

- A letter that responds to each point raised by the editor and reviewer(s). You should upload this letter as a separate file labeled 'Response to Reviewers'.
- A marked-up copy of your manuscript that highlights changes made to the original version. You should upload this as a separate file labeled 'Revised Manuscript with Track Changes'.
- An unmarked version of your revised paper without tracked changes. You should upload this as a separate file labeled 'Manuscript'.

Guidelines for resubmitting your figure files are available below the reviewer comments at the end of this letter.

We look forward to receiving your revised manuscript.

Kind regards,

Md. Alamgir Hossain, M.S.S., M.P.H.

Academic Editor

PLOS Global Public Health

Journal Requirements:

1. Please amend your detailed Financial Disclosure statement. This is published with the article. It must therefore be completed in full sentences and contain the exact wording you wish to be published.

i. State the initials, alongside each funding source, of each author to receive each grant. For example: "This work was supported by the National Institutes of Health (####### to AM; ###### to CJ) and the National Science Foundation (###### to AM)."

2. In the online submission form, you indicated that “The data that support the findings of this study are available from the corresponding author upon reasonable request. Due to the sensitive nature of the qualitative data and confidentiality requirements, full transcripts cannot be shared publicly.”.

All PLOS journals now require all data underlying the findings described in their manuscript to be freely available to other researchers, either

1. In a public repository,

2. Within the manuscript itself, or

3. Uploaded as supplementary information.

This policy applies to all data except where public deposition would breach compliance with the protocol approved by your research ethics board. If your data cannot be made publicly available for ethical or legal reasons (e.g., public availability would compromise patient privacy), please explain your reasons by return email and your exemption request will be escalated to the editor for approval. Your exemption request will be handled independently and will not hold up the peer review process, but will need to be resolved should your manuscript be accepted for publication. One of the Editorial team will then be in touch if there are any issues.

If the reviewer comments include a recommendation to cite specific previously published works, please review and evaluate these publications to determine whether they are relevant and should be cited. There is no requirement to cite these works unless the editor has indicated otherwise.

Additional Editor Comments (if provided):

[Note: HTML markup is below. Please do not edit.]

Reviewers' comments:

Reviewer's Responses to Questions

**Comments to the Author**

1. Does this manuscript meet PLOS Global Public Health’s publication criteria? Is the manuscript technically sound, and do the data support the conclusions? The manuscript must describe methodologically and ethically rigorous research with conclusions that are appropriately drawn based on the data presented.

Reviewer #1: Partly

Reviewer #2: Yes

**********

2. Has the statistical analysis been performed appropriately and rigorously?

Reviewer #1: Yes

Reviewer #2: Yes

**********

3. Have the authors made all data underlying the findings in their manuscript fully available (please refer to the Data Availability Statement at the start of the manuscript PDF file)?

The PLOS Data policy requires authors to make all data underlying the findings described in their manuscript fully available without restriction, with rare exception. The data should be provided as part of the manuscript or its supporting information, or deposited to a public repository. For example, in addition to summary statistics, the data points behind means, medians and variance measures should be available. If there are restrictions on publicly sharing data—e.g. participant privacy or use of data from a third party—those must be specified.

Reviewer #1: Yes

Reviewer #2: Yes

**********

4. Is the manuscript presented in an intelligible fashion and written in standard English?

PLOS Global Public Health does not copyedit accepted manuscripts, so the language in submitted articles must be clear, correct, and unambiguous. Any typographical or grammatical errors should be corrected at revision, so please note any specific errors here.

Reviewer #1: Yes

Reviewer #2: Yes

**********

5. Review Comments to the Author

Please use the space provided to explain your answers to the questions above. You may also include additional comments for the author, including concerns about dual publication, research ethics, or publication ethics. (Please upload your review as an attachment if it exceeds 20,000 characters)

Reviewer #1: Digital health services play an important role in equitable health care access in underserved areas. This is an important topic, looking at the barriers and facilitators to implementing tele psychotherapy in the global south.

Major concerns:

1. While the authors’ state that comparative assessments into in person and tele health services are scarce, there have been many papers exploring this area since the COVIC pandemic. For example, there have been publications on this area in PLOS digital health and PLOS GPH journals.

How is this study adding to the existing literature in this field?

https://journals.plos.org/digitalhealth/article?id=10.1371/journal.pdig.0000398

https://journals.plos.org/digitalhealth/article?id=10.1371/journal.pdig.0000934

https://journals.plos.org/globalpublichealth/article?id=10.1371/journal.pgph.000502

2. A more in-depth literature review of papers on this topic is required, and the authors are requested to elaborate how their findings add to or differ from existing data from other developing countries. While a few papers have been referenced in the discussion section, there is a huge body of work in this area, and a more detailed justification of this work is required.

Many of the findings of this study: for example, low income, poor internet access, or lack of govt support being barriers to engagement can be readily anticipated as barriers in a resource limited setting and have been demonstrated in prior research in similar settings worldwide.

3. The conclusions appear ambiguous and vague, with lack of specific results or implications.

Minor concerns:

3. While the authors have compared engagers vs non engagers in each group (tele health vs in person), were there common characteristics between engagers in both groups? And between the non engagers in both groups? This is important to identify factors that influence health care uptake regardless of the intervention used.

Reviewer #2: Overall Assessment

Topic is timely, policy-relevant, and of substantial global health interest. The integration of implementation science (CFIR) with qualitative inquiry is commendable. However, the manuscript presents conceptual inconsistencies, methodological ambiguities, structural issues, and interpretational concerns that require careful revision prior to publication.

Abstract

The abstract states “inductive thematic analysis,” whereas the Methods section later specifies deductive thematic analysis guided by CFIR. This contradiction must be resolved.

Precise engagement percentages (e.g., 61.69%, 10.27%) are presented without explanation of their analytical role within a phenomenological qualitative study. The distinction between RCT outcomes and qualitative findings should be clarified.

Introduction

The same numerical comparison between TSP and SMHS engagement appears multiple times. This risks redundancy and weakens narrative flow.

Statements such as Uganda ranking among “top countries with high depression rates” should be cautiously framed and directly supported by high-quality epidemiological sources.

The introduction justifies the need for teletherapy using the context of COVID-19 and resource shortages but overlooks temporal vulnerability—youth need support during specific vulnerable hours when clinics are closed. Adding this temporal aspect, supported by a recent study, fills this gap by establishing teletherapy as the only solution capable of reaching youth during these specific high-risk windows. Write below phrase with recent literature as suggested to strengthen the gap..

"Evidence on temporal patterns of suicide attempts reveals that vulnerability peaks during specific hours, precisely when traditional in-person mental health facilities are often closing or understaffed [Sheikh et al., 2024 Elsevier, https://doi.org/10.1016/j.bbii.2024.100072 ]. This temporal gap underscores that flexible interventions like tele-support psychotherapy (TSP) are not merely convenient alternatives but potentially life-saving services capable of reaching individuals during these identified high-risk windows."

Methods

The manuscript describes the study as: Phenomenological qualitative study, Guided by CFIRm Embedded within an RCT

Phenomenology is inherently inductive and experience-centered, whereas CFIR-based coding is typically deductive and framework-driven. The epistemological compatibility should be justified. Clarify whether the study is truly phenomenological or a framework-guided qualitative descriptive study.

Earlier: “inductive thematic analysis” and later: “deductive thematic analysis guided by CFIR”

Both cannot simultaneously be true without methodological clarification.

The sampling logic lacks sufficient detail: How many KIIs? How many FGDs per subgroup? How were engagers vs non-engagers balanced?

The statement: “By the fourth FGD data saturation was reached” is acceptable but requires operational definition of saturation.

Results

Major Concern is Quantitative Tables in Qualitative Study

Tables 1 and 2 present statistical hypothesis testing (t-tests, χ² tests, p-values), which is inconsistent with the stated qualitative phenomenological design. Either integrate as a mixed-methods design Or relocate statistical comparisons to supplementary material

“Marginally significant difference (p=0.055)” Strictly speaking, this is not statistically significant and should be framed cautiously.

Large gender disparities (female vs male engagement) are important but insufficiently explored. Potential sociocultural explanations should be considered.

Certain barriers (e.g., phone loss, network instability) could be analytically distinguished between intervention vs structural poverty.

Discussion

The discussion is comprehensive but would benefit from greater analytical discipline and reduced narrative drift.

Several themes are reiterated multiple times (income, social support, poverty). Condensation would improve impact.

The discussion occasionally implies causal inference (e.g., income “facilitates engagement”), which is not appropriate for qualitative design. Prefer like “Income appeared to influence engagement…”

Statements linking anti-LGBTQ+ legislation to engagement barriers are important but require careful evidentiary framing to avoid overgeneralization.

Although CFIR guided analysis, the discussion is not fully structured around CFIR domains. Stronger framework integration would enhance theoretical contribution.

“Our findings echo what others have seen…” Should be formalized.

Conclusion

Suggestion for Strengthening: “These findings highlight the necessity of addressing structural socioeconomic constraints alongside innovation-specific barriers when implementing digital mental health interventions in low-resource settings.”

**********

6. PLOS authors have the option to publish the peer review history of their article (what does this mean?). If published, this will include your full peer review and any attached files.

**Do you want your identity to be public for this peer review?**  If you choose “no”, your identity will remain anonymous but your review may still be made public.

For information about this choice, including consent withdrawal, please see our Privacy Policy.

Reviewer #1: No

Reviewer #2: No

**********

[NOTE: If reviewer comments were submitted as an attachment file, they will be attached to this email and accessible via the submission site. Please log into your account, locate the manuscript record, and check for the action link "View Attachments". If this link does not appear, there are no attachment files.]

-->Figure Resubmissions:

While revising your submission, we strongly recommend that you use PLOS’s NAAS tool (https://ngplosjournals.pagemajik.ai/artanalysis) to test your figure files. NAAS can convert your figure files to the TIFF file type and meet basic requirements (such as print size, resolution), or provide you with a report on issues that do not meet our requirements and that NAAS cannot fix.

After uploading your figures to PLOS’s NAAS tool - https://ngplosjournals.pagemajik.ai/artanalysis, NAAS will process the files provided and display the results in the "Uploaded Files" section of the page as the processing is complete. If the uploaded figures meet our requirements (or NAAS is able to fix the files to meet our requirements), the figure will be marked as "fixed" above. If NAAS is unable to fix the files, a red "failed" label will appear above. When NAAS has confirmed that the figure files meet our requirements, please download the file via the download option, and include these NAAS processed figure files when submitting your revised manuscript.

Attachment Submitted filename: Comments.docx

# Author comment: Author response to Decision Letter 1

**DOI:** 10.1371/journal.pgph.0006657.r002

18 Apr 2026

Attachment Submitted filename: Response to Reviewers.docx

# Peer review: Decision Letter 1

**DOI:** 10.1371/journal.pgph.0006657.r003

**Authors:** Md. Alamgir Hossain

28 May 2026

Barriers and Facilitators to Tele-Support Psychotherapy Versus Standard In-Person Mental Health Services for Youth (15-30 Years) with Depression in Kampala District, Uganda.

PGPH-D-25-03671R1

Dear Dr. Etheldreda Nakimuli-Mpungu,

We are pleased to inform you that your manuscript 'Barriers and Facilitators to Tele-Support Psychotherapy Versus Standard In-Person Mental Health Services for Youth (15-30 Years) with Depression in Kampala District, Uganda.' has been provisionally accepted for publication in PLOS Global Public Health.

Before your manuscript can be formally accepted you will need to complete some formatting changes, which you will receive in a follow up email. A member of our team will be in touch with a set of requests.

Please note that your manuscript will not be scheduled for publication until you have made the required changes, so a swift response is appreciated.

IMPORTANT: The editorial review process is now complete. PLOS will only permit corrections to spelling, formatting or significant scientific errors from this point onwards. Requests for major changes, or any which affect the scientific understanding of your work, will cause delays to the publication date of your manuscript.

If your institution or institutions have a press office, please notify them about your upcoming paper to help maximize its impact. If they'll be preparing press materials, please inform our press team as soon as possible -- no later than 48 hours after receiving the formal acceptance. Your manuscript will remain under strict press embargo until 2 pm Eastern Time on the date of publication. For more information, please contact globalpubhealth@plos.org.

Thank you again for supporting Open Access publishing; we are looking forward to publishing your work in PLOS Global Public Health.

Best regards,

Md. Alamgir Hossain, M.S.S., M.P.H.

Academic Editor

PLOS Global Public Health

***********************************************************

Reviewer Comments (if any, and for reference):

Reviewer's Responses to Questions

**Comments to the Author**

1. If the authors have adequately addressed your comments raised in a previous round of review and you feel that this manuscript is now acceptable for publication, you may indicate that here to bypass the “Comments to the Author” section, enter your conflict of interest statement in the “Confidential to Editor” section, and submit your "Accept" recommendation.

Reviewer #3: All comments have been addressed

Reviewer #4: All comments have been addressed

**********

2. Does this manuscript meet PLOS Global Public Health’s publication criteria? Is the manuscript technically sound, and do the data support the conclusions? The manuscript must describe methodologically and ethically rigorous research with conclusions that are appropriately drawn based on the data presented.

Reviewer #3: Yes

Reviewer #4: Yes

**********

3. Has the statistical analysis been performed appropriately and rigorously?

Reviewer #3: N/A

Reviewer #4: Yes

**********

4. Have the authors made all data underlying the findings in their manuscript fully available (please refer to the Data Availability Statement at the start of the manuscript PDF file)?

The PLOS Data policy requires authors to make all data underlying the findings described in their manuscript fully available without restriction, with rare exception. The data should be provided as part of the manuscript or its supporting information, or deposited to a public repository. For example, in addition to summary statistics, the data points behind means, medians and variance measures should be available. If there are restrictions on publicly sharing data—e.g. participant privacy or use of data from a third party—those must be specified.

Reviewer #3: Yes

Reviewer #4: Yes

**********

5. Is the manuscript presented in an intelligible fashion and written in standard English?

PLOS Global Public Health does not copyedit accepted manuscripts, so the language in submitted articles must be clear, correct, and unambiguous. Any typographical or grammatical errors should be corrected at revision, so please note any specific errors here.

Reviewer #3: Yes

Reviewer #4: Yes

**********

6. Review Comments to the Author

Please use the space provided to explain your answers to the questions above. You may also include additional comments for the author, including concerns about dual publication, research ethics, or publication ethics. (Please upload your review as an attachment if it exceeds 20,000 characters)

**Reviewer #3:**  Thank you for the opportunity to review this revised manuscript. The authors have adequately addressed all comments and suggestions raised during the peer review process, including clarifications related to the methodological framework, contextualization of the findings, and strengthening of the discussion and limitations sections. The revised version is substantially improved, well-structured, and provides an important contribution to the literature on digital mental health interventions for youth in low-resource settings. I have no further concerns and recommend acceptance for publication. I look forward to seeing this important study published in PLOS Global Public Health.

**Reviewer #4:**  (No Response)

**********

7. PLOS authors have the option to publish the peer review history of their article (what does this mean?). If published, this will include your full peer review and any attached files.

**Do you want your identity to be public for this peer review?**  If you choose “no”, your identity will remain anonymous but your review may still be made public.

For information about this choice, including consent withdrawal, please see our Privacy Policy.

Reviewer #3: **Yes:**  Monica Malta

Reviewer #4: No

**********
